# The Role of Milk Oligosaccharides in Enhancing Intestinal Microbiota, Intestinal Integrity, and Immune Function in Pigs: A Comparative Review

**DOI:** 10.3390/biology13090663

**Published:** 2024-08-26

**Authors:** Alexa Gormley, Yesid Garavito-Duarte, Sung Woo Kim

**Affiliations:** Department of Animal Science, North Carolina State University, Raleigh, NC 27695, USA; agormle@ncsu.edu (A.G.); yrgaravi@ncsu.edu (Y.G.-D.)

**Keywords:** intestinal microbiota, immunomodulation, nursery pigs, milk coproducts, milk oligosaccharides, suckling pigs

## Abstract

**Simple Summary:**

Young pigs face many challenges associated with weaning, including disruption to a healthy and robust intestinal microbiota, leaving them vulnerable to infection with enteric pathogens. Modern farming practices wean piglets at a younger age than they would outside of artificial rearing, and therefore their immature intestines may not be equipped to handle the challenges associated with weaning. Milk oligosaccharides are prebiotics naturally found in the colostrum and milk of mammals and have been shown to improve intestinal health in young mammals. Milk oligosaccharides can encourage the development of mature intestines, protect the host from pathogens, and modulate host immune responses. Milk coproducts from bovine sources are commonly utilized in nursery pig feeds and can be a source of milk oligosaccharides past the suckling period. However, bovine milk coproducts have a different oligosaccharide composition when compared to those found in porcine milk. This review summarizes the benefits associated with specific milk oligosaccharides for suckling and nursery pigs.

**Abstract:**

The objective of this review was to identify the characteristics and functional roles of milk coproducts from human, bovine, and porcine sources and their impacts on the intestinal microbiota and intestinal immunity of suckling and nursery pigs. Modern pig production weans piglets at 3 to 4 weeks of age, which is earlier than pigs would naturally be weaned outside of artificial rearing. As a result, the immature intestines of suckling and nursery pigs face many challenges associated with intestinal dysbiosis, which can be caused by weaning stress or the colonization of the intestines by enteric pathogens. Milk oligosaccharides are found in sow milk and function as a prebiotic in the intestines of pigs as they cannot be degraded by mammalian enzymes and are thus utilized by intestinal microbial populations. The consumption of milk oligosaccharides during suckling and through the nursery phase can provide benefits to young pigs by encouraging the proliferation of beneficial microbial populations, preventing pathogen adhesion to enterocytes, and through directly modulating immune responses. Therefore, this review aims to summarize the specific functional components of milk oligosaccharides from human, bovine, and porcine sources, and identify potential strategies to utilize milk oligosaccharides to benefit young pigs through the suckling and nursery periods.

## 1. Introduction

In humans, the role of milk oligosaccharides (MO) was first discovered when researchers worked to understand clinical and physiological differences between breastfed and bottle-fed infants [1]. It was first identified that microbial populations in the feces of infants fed with breastmilk were different from those of infants that were formula fed [2], and this later contributed to the discovery of factors in the whey fraction of breastmilk that had a growth-promoting effect on *Bifidobacterium bifidus*, and later this factor was identified as an oligosaccharide [3,4]. At this time, it was established that MO have a positive influence on microbial communities in the intestines of infants. However, the other functional properties still required investigation. Since then, much of the research surrounding MO has been done in humans, as humans have a relatively high concentration and diversity of MO when compared with other mammals, although MO are still of great importance to the growth and development of all mammalian species, including pigs [5,6].

In pigs, the MO concentration in milk is less than that of human milk but has a similar composition. According to Albrecht et al. [7], the MO present in porcine milk contain a higher percentage of certain components like neutral oligosaccharides when compared with humans, but the overall composition is more similar to humans than to other mammals. Although MO composition varies throughout lactation, the reduced concentration and diversity of porcine MO (PMO) naturally occurring in colostrum and milk is related to the prevention of pathogen binding to the intestinal epithelium and the stimulation of beneficial bacterial growth in the neonatal gut [8]. As piglets are born with immature intestines and are at risk of intestinal infection and dysbiosis, research on the function of naturally occurring PMO during lactation and the application of non-porcine MO during the nursery period could reveal the benefits of a targeted selection of MO sources for pigs. Therefore, the purpose of this review is to characterize the functional roles of naturally present PMO; identify the differences in MO from human, bovine, and porcine sources; and discuss the practical applications of MO for pigs beyond the suckling period.

## 2. Characterization of Milk Oligosaccharides

**Figure 1:**
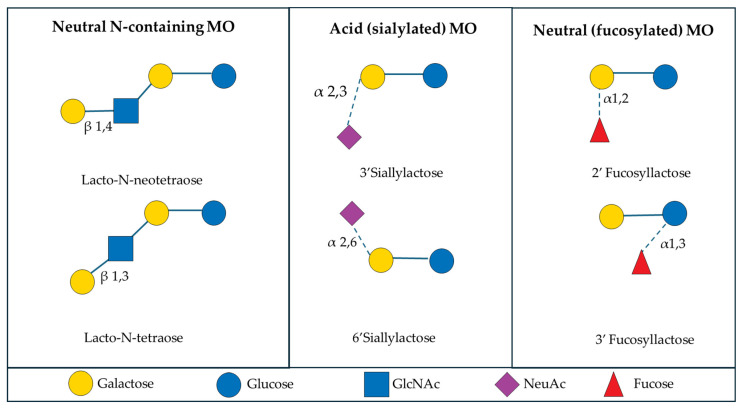
Examples of the structures of six common milk oligosaccharides, grouped into three primary subclasses. Abbreviations: GlcNAc: N-acetylglucosamine; NeuAc: N-acetylneuraminicacid.

Lactose and MO are the predominant carbohydrate sources in mammalian milk [9]. Milk oligosaccharides are considered non-nutritive bioactive factors, or prebiotics, because they cannot be effectively hydrolyzed by mammalian enzymes due to the arrangement of their linkages, but they still provide benefits to the host through their interaction with the intestinal microbiome [10,11,12]. Typically MO are composed of the monosaccharides glucose, galactose, N-acteyl-glucosamine, fucose, and sialic acid [13]. Most MO contain lactose, a disaccharide composed of glucose and galactose, at the reducing end and the structure will be extended with the addition of lacto-N-biose I or lactosamine to form the base chain [14]. From the base chain, further modification through the inclusion of fucose and sialic acid in branched or linear side chains allows for vast complexity and uniqueness across all MO [13,15] (Figure 1). In addition to the diversity of MO, the composition of each type of MO varies considerably between mammalian species, as will be further discussed in this review.

### Characterization of Milk Oligosaccharides from Human, Bovine, and Porcine Sources

The colostrum and milk of mammals is a rich source of oligosaccharides and MO composition has variance between individuals that is influenced by external factors like genetics, maternal age, stage of lactation, and diet [16,17,18,19,20,21]. There are six common MO, Lacto-N-neotetraose, Lacto-N-tetraose, 3′Sialyllactose, 6′Sialyllactose, 2′Fucosyllactose, and 3′Fucosyllactose, that fit within three general subclasses of MO: neutral N-containing MO, acid (sialylated) MO, and neutral (fucosylated) MO. Generally, neutral N-containing MO have nitrogen-containing groups, like N-acetylglucosamine, but no fucose or sialic acid residues [22]. Sialylated MO contain sialic acid residues, usually attached at the terminal end of the oligosaccharide chain, whereas fucosylated MO contain fucose residues, but lack sialic acid or nitrogen-containing groups [22]. Milk oligosaccharides can also be classified by their chain type, with type I MO containing Galβ1–3GlcNAc units at their terminal end, whereas the type II MO contain Galβ1-4GlcNAc units at their terminal end [23]. There is considerable variety in the structure of MO present across mammalian species, and there are instances in which some MO can contain structures that belong to more than one primary subclass. However, for the purpose of this review, the focus will remain on the six common MO and the three major subclasses of MO, as previously described.

**Figure 2:**
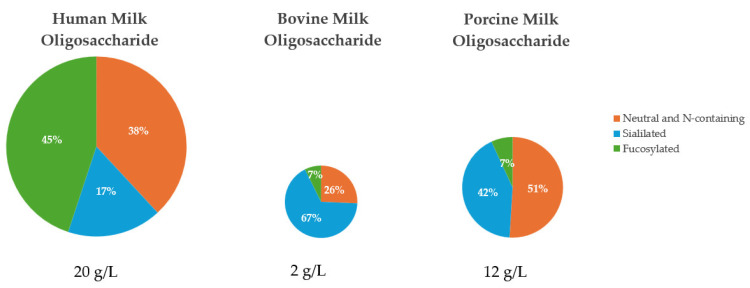
Composition of the six common milk oligosaccharides grouped into their respective major subclasses of MO found in pooled early milk from human [25,26], bovine [7,29], and porcine sources [8,32,34]. Human milk contains the greatest concentration and diversity of MO of all studied mammals to date [11].

The amount and relative abundance of the three major subclasses of MO varies considerably in human, bovine, and porcine milk (Figure 2). Human milk contains the greatest concentration and diversity of MO of all mammals, with human milk containing approximately 5 to 20 g/L of unbound oligosaccharides [24] and over 200 unique human milk oligosaccharides (HMO) structures [19]. The amount of HMO typically exceeds the total protein content of milk and is 100 to 1000 times greater than the concentration of MO found in bovine milk [19]. The fucosylated and neutral N-containing MO are the most abundant in human pooled colostrum and milk, at approximately 45% and 38%, respectively [25,26].

In contrast, bovine MO (BMO) are significantly less concentrated than HMO, as identified by liquid chromatography-mass spectrometry, which found the total concentration of MO to be 100 mg/L to 2 g/L in bovine milk [27]. In the case of BMO, approximately 25 unique MO structures have been identified [28]. The most abundant MO in early bovine milk are sialylated and neutral N-containing MO, making up approximately 67% and 26%, respectively [7,29]. The dominant structures present in BMO are 3′-sialyllactose (3′SL) and 6′-sialyllactose (6′SL), and these are found as a large percentage of the BMO pool [27,30]. Fong et al. [28] reported a range of 47 to 55 μg/mL for 3′ SL and 3.6 to 9.6 μg/mL for 6′SL in BMO.

Less is known about the composition of PMO than HMO and BMO, but in general, the composition of PMO are thought to be more similar to HMO than BMO. However, the overall concentration of MO is less in pigs than it is in humans [7]. In pigs, there are approximately 55 unique structures identified to date [20], with the total concentration of MO to be 6 to 12 g/L in porcine milk [31]. According to several authors [7,8,32], the neutral N-containing MO are the most abundant MO in early milk at approximately 51%, followed by sialylated MO at 42%, which are highest in colostrum and decrease throughout lactation. Interestingly, Albrecht et al. [7] reported that porcine milk contained approximately 20% fucosylated MO, the highest of all domestic species investigated, despite fucosylated MO having the lowest relative abundance of all MO in porcine milk. At large, it could be assumed that porcine colostrum and milk should be more similar to that of human colostrum and milk, than bovine, due to the similarities between humans and pigs, especially in the gastrointestinal tract [8,33]. Across all three species, 3′SL and 6′SL are reported to be the primary common MO between human, bovine, and porcine colostrum [8]. Porcine milk was reported to contain 2′-fucosyllactose and type I MO in greater abundance than type II MO, similar to human milk [8]. It is important to note that although PMO and HMO are not identical, they share more similarities than between PMO and BMO. Notably, the similarities between PMO and HMO increase as lactation progresses as a result of PMO showing an increase in fucosylation and a decrease in sialylation throughout lactation [8].

## 3. Functional Role of Milk Oligosaccharides

In the wild, a piglet would gradually be weaned from its mother at approximately 10 to 12 weeks of age, but commercially, piglets are weaned at approximately 3 to 4 weeks of age [35]. Therefore, the quantity and quality of colostrum and milk consumed during the lactation period is of great interest to pig producers. Like other mammals, piglets are born with immature intestines, and piglets specifically are born with a naïve immune system, as maternal immunoglobulins cannot pass through the placenta to interact with piglets in utero [36]. This, combined with the stressors associated with early weaning, leaves the intestines vulnerable to enteric pathogens and intestinal dysbiosis, which can disrupt intestinal development, damage existing intestinal tissue, and negatively affect the intestinal microbiota, thereby decreasing growth and increasing rates of morbidity and mortality in the post-weaning period [37,38]. The intestines of pigs will continue to mature through the post-weaning period, indicating that the inclusion of MO could continue to provide functional effects when the pigs are fed during the nursery phase.

**Figure 3:**
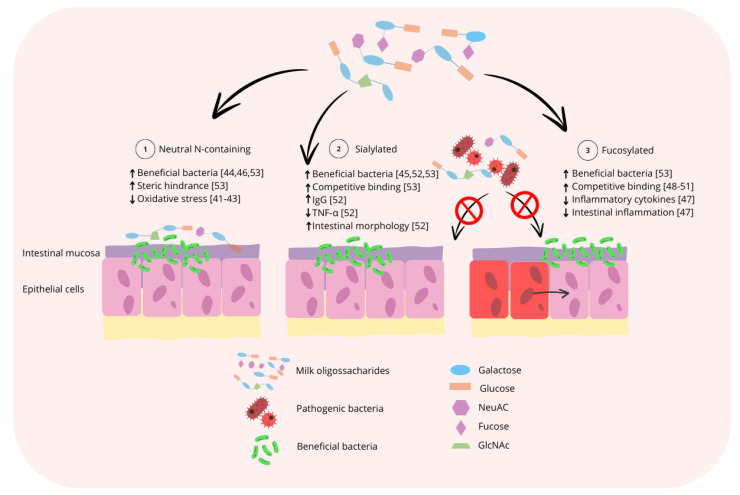
Summary of the generalized mode of action for the three major subclasses of MO. 1: Neutral N-containing MO could increase populations of potentially beneficial bacteria, such as *Bifidobacterium* spp., prevent pathogen binding to the host epithelium by steric hinderance, thereby reducing oxidative stress and improving intestinal morphology. 2: Sialylated MO could increase populations of potentially beneficial bacteria, prevent pathogen binding by competitive binding to pathogens, thereby increasing serum IgG and decreasing serum TNF-α. 3: Fucosylated MO increase the populations of potentially beneficial bacteria and prevent pathogen binding by competitive binding to pathogens, thereby reducing the secretion of inflammatory cytokines and intestinal inflammation [41,42,43,44,45,46,47,48,49,50,51,52,53].

During the nursery phase, milk coproducts are commonly utilized due to the high digestibility of lactose when compared with the complex carbohydrates found in cereal grains and the high levels of lactase activity still present from the suckling period [39,40]. Milk coproducts are primarily derived from bovine sources, which have a different composition of MO when compared with the composition of PMO. As a result, the use of bovine products during the nursery phase may not provide pigs with the optimal composition of MO to support intestinal and immune system development and the maintenance of intestinal microbiota symbiosis, which is of significant financial interest to pig producers. By identifying the primary functional roles of MO for pigs, it could also be identified which functional MO are missing from bovine sources. A visual summary of the generalized mode of action of the three major subclasses of MO as it relates to support of the intestinal microbiota and intestinal immune system is displayed in Figure 3.

### 3.1. Influence on the Intestinal Microbiota

The establishment of the intestinal microbiota is pivotal in the development of the intestinal immune system and maintenance of the intestinal barrier, ultimately influencing the growth and health of pigs [37,54,55]. Both the luminal and mucosa-associated microbiota have an influence on the digestion and absorption of nutrients, in addition to interactions with the host immune system [56,57]. Therefore, feedstuffs that can have a positive influence on the intestinal microbiota warrant consideration for use in a feeding program for nursery pigs. As previously mentioned, MO have a prebiotic function in the intestine of mammals, primarily influencing the intestinal microbiota.

According to Frese et al. [58], the fecal microbiota populations stabilize shortly after birth in pigs, with the general composition of the microbiota remaining consistent for the first three weeks of life, dominated by a “milk-oriented microbiota.” The milk-oriented microbiota of the large intestine consists of populations more adept at utilizing the components of milk, including MO. For example, Bacteroides have been reported to effectively utilize MO [59,60], which is in agreement with the high relative abundance of Bacteroidaceae observed in the fecal microbiome of suckling pigs [58]. Similarly Enterobacteriaceae was observed to be a dominant family in the fecal microbiota of suckling pigs [58], a family with many roles in the maintenance of intestinal health, with some species causing enteric disease and others being a normal part of intestinal populations [61]. Enterobacteriaceae are able to effectively consume sialic acid, which is highly available in porcine colostrum and decreases throughout lactation, and is reflected in the decrease in the relative abundance of Enterobacteriaceae after weaning [8,62]. Although the milk-oriented microbiota is well equipped to utilize the nutritive components of milk, suckling pigs also have the lowest microbial diversity in the gastrointestinal tract of any age group, which contributes to their susceptibility to enteric infection from bacterial populations found in the intestines [63,64,65,66].

Milk oligosaccharides can selectively stimulate the growth of certain bacterial populations, and most notably are known to have a bifidogenic effect [41]. Select Bifidobacterium spp. have been shown to reduce oxidative stress, improve intestinal morphology, and increase the relative abundance of generally beneficial bacterial populations in the luminal environment of both the small and large intestine, thereby reducing incidences of diarrhea and improving growth performance in nursery pigs [42,43]. Typically, Bifidobacterium are utilized as a direct-feed probiotic. However, targeted use of MO could increase the populations of Bifidobacterium in the intestines without direct supplementation. Notably, Bifidobacterium have been shown to be responsive to many of the most common members of the three subclasses of MO, including neutral N-containing MO like lacto-N-tetraose (LNT) and lacto-N-neotetraose (LNnT), neutral MO like *2′*-fucosyllactose, and sialylated MO like *3′*SL and *6′*SL [44,45,54]. A recent study investigated the effects of the supplementation of BMO in the traditional diets of Malawian infants on the growth and health outcomes in infants whose growth was severely stunted [67]. In this study, newborn piglets were utilized and colonized with the bacterial strains isolated from the stunted infants, and it was found that the supplementation of sialylated BMO improved growth and shifted the fecal microbiota to favor nutrient utilization. Similarly, it was found that use of sialylated MO could impact the luminal microbiota of both the distal and proximal colon, increasing populations generally regarding as beneficial and decreasing populations of potentially pathogenic populations, such as Enterobacteriaceae, in pigs fed diets supplemented with sialylated MO, although no improvements to growth performance were observed in this study [68].

Beyond the suckling period, MO can continue to support the development of the mature microbiome in nursery pigs. During the suckling period, the genus Prevotella is found in low relative abundance in the lumen [58], but the relative abundance increases dramatically as pigs age and begin to consume plant-derived carbohydrates, which is consistent with the feeding preference of Prevotella spp. [69,70]. This increase in Prevotella coincides with the replacement of the previously more abundant Bacteriodes during the suckling period, specifically in the ileal digesta [71]. In commercial pig production, lactose products are maintained in the diet through the nursery phase, despite the activity of lactase decreasing by 60% to 80% in the small intestine of nursery pigs at 8 days post-weaning [72]. Lactose in the diet beyond the enzymatic capabilities of the pig could result in excessive lactose fermentation and increase the incidence of diarrhea and alteration of intestinal motility [73,74], which could compound with pre-existing enteric infections [75]. Similarly, pathogenic bacteria have a decreased capability to utilize MO, allowing for the more effective MO users to dominate over enteric pathogens [76]. Furthermore, the metabolism of MO by Bifidobacterium, for example, results in the production of organic acids that create an acidic environment that is inhospitable for many pathogenic bacteria [77].

In general, the support of a diverse and robust intestinal microbiota is the key to maintaining the growth and health of pigs, especially through the weaning period. Milk oligosaccharides provide unique prebiotic support to the intestinal microbiota, diversifying microbial populations and leading to potential positive influences on intestinal morphology, digestive and absorptive capacities, and intestinal immune system interactions, while simultaneously preventing the development of an environment that favors the growth of enteric pathogens.

### 3.2. Immunomodulatory Properties

It is well understood that MO have immunomodulatory properties, with one of the earliest works related to the immunomodulatory properties of MO identifying the influence of human breast milk on the later development of eczema [78]. More recently, several other human studies have identified the influence of breast milk on reducing future allergenic reactions when compared to formula-fed infants [79,80,81,82]. Milk oligosaccharides serve a role in the immune system by modulating interactions between host immune cells, preventing the adherence of pathogenic bacteria to intestinal epithelium, or by reducing inflammation [83,84]. To date, few studies have investigated the mechanisms of action associated with MO and their immunoregulatory properties in the intestines of pigs, especially when investigating the benefits of different MO sources. However, the expanse of research related to MO and immunity in human infants suggests that similar results may be achievable in a porcine model, and thus warrants further investigation.

Milk oligosaccharides can directly influence the host immune response. For example, in a cellular model, it was found that treatment with HMO mediated intestinal inflammation associated with lipopolysaccharides (LPS) from pathogenic Escherichia coli, due to the presence of *2′*FL [46]. The use of *2′*FL at 2 mg/mL was shown to decrease the secretion of the cytokine IL-8 in T84 cells by 45% and the suppression was found to be dose-dependent up to 4 mg/mL *2′*FL, suggesting that sufficient dietary *2′*FL could reduce intestinal inflammation associated with Escherichia coli [46]. It is important to note that suppression of IL-8 was not maintained after the conclusion of the infection, suggesting that the inhibition of IL-8 is directly related to Escherichia coli infection. Similarly, it was found that infants fed formula supplemented with *2′*FL had lower plasma levels of inflammatory cytokines than infants fed non-supplemented formula, and had cytokine levels more similar to the breast-fed infants, emphasizing the importance of MO in mediation of the host immune response in young mammals [47]. In general fucosylated MO have been cited to prevent the adhesion of several common enteric pathogens such as Helicobacter pylori [48], Escherichia coli [49,50], and Campylobacter jejuni [51]. Milk coproducts derived from bovine sources have a much lower concentration of fucosylated MO when compared with human or porcine milk [85]. As such, inclusion of bovine milk coproducts in diets for nursery pigs may not provide the benefits associated with prevention of pathogen adhesion associated with fucosylated MO. Infection with enteric pathogens is a significant concern during the weaning period. Therefore, supplementation of fucosylated MO may benefit nursery pigs beyond what can be provided by bovine milk coproducts.

Despite the lack of fucosylated MO in bovine milk coproducts, there are still benefits associated with the presence of BMO in the diets of piglets. For example, it was found that the oral administration of sialyllactose to suckling piglets increased serum IgG levels and decreased serum TNF-α, indicating an association between the dietary inclusion of MO and the immune system [52]. Further research suggests that sialylated MO may inhibit the proliferation of intestinal epithelial cells and encourage cell differentiation during early life due to their interactions with the epidermal growth factor receptor [86]. Bacterial surfaces contain glycoconjugates similar to those found in host cell membranes, allowing them to compete for binding sites. The inclusion of *3′*SL and *6′*SL can inhibit or reduce the in vitro adhesion of pathogens such as Salmonella, Escherichia coli, Vibrio cholerae [53], and rotavirus [87]. In general, sialylated MO have the ability to inhibit pathogen adhesion to the intestines, as the structure of sialylated MO has the ability to be recognized by both the lectin receptors of the intestinal epithelium and of the potential pathogens [88]. Hester et al. [87] suggest that the neutral N-containing MO LNnT, which are also a more prominent component of BMO, could generate anti-inflammatory mediators that suppress T cell and inflammatory responses in vitro, and this is in agreement with the results of an in vivo study [89]. In the in vivo study, it was found that the HMO influenced the host protective immunity by stimulating a balanced response from the T helper type I cells, marked by higher levels of interferon-gamma, as well as an enhanced anti-inflammatory response associated with IL-10 in the ileum [89]. Additionally, the inclusion of LNnT produces larger amounts of short chain fatty acids (SCFA), which could potentially inhibit the replication of some viruses, such as rotavirus [87].

### 3.3. Impact on Intestinal Development, Nutrient Absorption, and Growth

Given the positive effects on the intestinal microbiota and immunomodulatory properties associated with the use of MO in the diet, it could be assumed that there may also be improvements to nutrient absorption and growth performance. Intestinal morphology can be an indicator of improved nutrient absorption, leading to improved growth performance. Improvements in intestinal morphology have been observed in some studies utilizing MO, suggesting that MO can encourage proper intestinal development and enhanced absorptive capacities. For example, in a study conducted by Li et al. [52], the oral inclusion of sialyllactose improved the growth performance of suckling pigs by 7.5%. The same study also reported that sialyllactose supplementation increased villus height and the villus-to-crypt depth ratio in the jejunum, suggesting an increased absorptive surface area and a higher rate of epithelial cell turnover in the intestine [52]. Another study observed that pigs fed a formula supplemented with *2′*FL had a tendency to have an increased ileal crypt depth and area, as well as elevated ileal sucrase activity in response to *2′*FL, which is a marker often related to epithelial cell differentiation [53,90]. Despite these improvements, there were no observed improvements to overall growth performance [90].

Despite improvements to intestinal morphology, the use of MO in diets for pigs has had inconsistent effects on growth performance, despite numerous studies linking the presence of HMO with appropriate growth in human models [47,91,92,93,94]. Supplementation of sialyllactose at different doses (130 mg/L, 380 mg/L, and 760 mg/L) in neonatal pigs supported normal growth, and did not affect the intestinal growth, morphology, goblet cell numbers, disaccharidase activity, fecal consistency, or cecal and colonic digesta pH [95]. Similarly, in an experiment conducted by Golden et al. [96] newborn pigs fed with milk replacer supplemented with *3′*-SL and *6′*-SL did not exhibit any differences in growth performance when compared with the control group, which could be related with the main function of sialic acid present in sialyllactose to act as a neuronal or microbial support [68]. Furthermore, the supplementation of *3′*SL and *6′*SL did not impact blood biomarkers or intestinal histomorphology [96]. In contrast, a study examining the dose-response and supplemental effects of increasing levels of whey permeate displayed some improvements to growth performance, potentially related to increasing amounts of MO in the diets [40]. In this study, the increased growth performance could be related to both the increase in total MO consumed and the increase in whey permeate included as a highly digestible feed ingredient [40]. Similarly, this study observed positive changes to the mucosa-associated microbiota in the jejunum, increased intestinal immune responses, and increased enterocyte proliferation, which could be attributed to the functional roles of MO [40].

As previously discussed, the influence of MO on the intestinal microbiota can have an impact on growth performance due to both the direct interactions between the host and the intestinal microbiota and the indirect impact of metabolites produced by the intestinal microbiota of the host. For example, it has been theorized that the use of MO can improve SCFA production by encouraging the growth of certain bacterial populations, namely Bifidobacterium [97,98]. The increased production of SCFA could result in additional energy for the host, which could improve growth performance [77,99,100,101]. Aside from bodyweight changes, the presence of MO could improve the growth and development of other organ systems, such as the skeletal system and the nervous system, as observed in humans [67,93,102,103], mice [104,105,106,107], and pigs [90,108,109]. Although studies reveal inconsistent results of the effect of MO on growth performance in pigs, the benefits associated with MO prove valuable to the overall health and growth of young pigs.

## 4. Conclusions

Milk oligosaccharides provide unique benefits to the intestinal health of young mammals. Due to rapid advances to pig production, suckling and nursery pigs face unique intestinal challenges as a result of weaning stress and environmental pathogens on the immature intestines and naïve immune system. The adverse effects of weaning on the intestines can have implications for piglet growth, morbidity, and mortality, ultimately having financial implications for the pig producer. Therefore, the continued use of milk oligosaccharides through the suckling and nursery periods may support the intestinal health of pigs through the weaning process. Milk coproducts from bovine sources are commonly utilized in nursery feeds. However, it is known that the composition of the milk oligosaccharides present in milk varies by species. Bovine milk tends to be higher in sialylated milk oligosaccharides than in milk of human or porcine origin. Benefits can be seen with the use of all milk oligosaccharides. However, benefits specific to the prevention of pathogen adhesion are more commonly associated with fucosylated milk oligosaccharides, which are present in much lower concentrations in bovine milk compared with human or porcine milk. As such, bovine milk coproducts may not provide the optimal benefits for nursery pigs. To date, few studies have been conducted examining the unique role of different sources of milk oligosaccharides for nursery pigs, but the current research suggests that further investigation into the use of targeted milk oligosaccharides could prove useful in improving the growth and health of nursery pigs. The use of milk oligosaccharides beyond the suckling period provides benefits to nursery pigs, and the use of specific milk oligosaccharides may provide targeted benefits related to the modulation of the intestinal microbiota, prevention of pathogen adhesion, and the modification of host immune responses.

## Data Availability

No new data were created or analyzed in this study. Data sharing is not applicable to this article.
